# High CD73 Expression Is Associated with Poor Prognosis in Biliary Tract Cancer Through Reduced Stromal Tumor-Infiltrating Lymphocytes

**DOI:** 10.3390/cancers18060975

**Published:** 2026-03-18

**Authors:** Shoya Shiratori, Kazumichi Kawakubo, Kanako C. Hatanaka, Takuma Kobayashi, Teppei Konishi, Yoshiki Shinomiya, Soichiro Oda, Shunichiro Nozawa, Hiroki Yonemura, Ryo Sugiura, Kazuaki Harada, Yoshitsugu Nakanishi, Takehiro Noji, Shinya Tanaka, Satoshi Hirano, Masaki Kuwatani, Yutaka Hatanaka, Naoya Sakamoto

**Affiliations:** 1 Department of Gastroenterology and Hepatology, Hokkaido University Hospital, North 14, West 5, Kita-ku, Sapporo 060-8648, Hokkaido, Japan; swan.0723.spr@gmail.com (S.S.); ryou99sugi@yahoo.co.jp (R.S.);; 2 Center of Development of Advanced Diagnostics, Hokkaido University Hospital, North 14, West 5, Kita-ku, Sapporo 060-8648, Hokkaido, Japan; kyanack@huhp.hokudai.ac.jp (K.C.H.);; 3 Biomy Inc., 3-8-3, Nihonbashi-honcho, Chuo-ku, Tokyo 103-0023, Japan; 4 Department of Gastroenterological Surgery II, Faculty of Medicine and Graduate School of Medicine, Hokkaido University, North 15, West 7, Kita-ku, Sapporo 060-8638, Hokkaido, Japansatto@med.hokudai.ac.jp (S.H.); 5 Division of Nursing, Faculty of Health Sciences, Hokkaido University, North 12, West 5, Kita-ku, Sapporo 060-0812, Hokkaido, Japan; 6 Department of Surgical Pathology, Hokkaido University Hospital, North 14, West 5, Kita-ku, Sapporo 060-8648, Hokkaido, Japan

**Keywords:** biliary tract cancer, CD73, infiltrating lymphocyte, stroma, digital pathology

## Abstract

**Background:** Biliary tract cancer (BTC) is an aggressive malignancy with limited therapeutic options and a poor prognosis. CD73 is upregulated under hypoxic conditions and promotes tumor progression. However, its clinical role in BTC and interaction with tumor-infiltrating lymphocytes (TILs) remain unclear. This study aimed to elucidate the association between CD73 expression and prognosis in BTC, as well as its impact on the tumor microenvironment (TME) and TILs. **Methods:** This retrospective study included 100 patients who underwent curative BTC surgery at Hokkaido University Hospital between 2018 and 2023. Formalin-fixed tumor specimens were analyzed using DeepPathFinder™ (biomy Inc., Tokyo, Japan), an AI-based digital pathology platform enabling objective quantification of CD73 expression and lymphocyte infiltration within tumoral (T) and stromal (S) compartments. Immunohistochemistry for CD3, CD8, Foxp3, and CD163 was used to identify T-cell subsets and macrophages. Associations between CD73, TIL subsets, and overall survival (OS) were assessed using the Kaplan–Meier, Cox regression, and Spearman correlation analyses. **Results:** High T-CD73 expression was associated with shorter OS (hazard ratio [HR] = 1.97, *p* = 0.041), whereas S-CD73 showed no prognostic relevance. Conversely, high S-TIL density was correlated with improved survival (HR = 0.49, *p* = 0.032). T-CD73 expression was negatively correlated with stromal CD3^+^ and CD8^+^ T-cell densities, indicating selective suppression of stromal cytotoxic T-lymphocyte (CTL) infiltration. No significant correlations were observed between Foxp3^+^ T cells and CD163^+^ M2 macrophages. **Conclusions:** CD73 upregulation in tumor cells impairs stromal CTL and TIL activity, leading to a poor prognosis. Spatial distribution, rather than total TIL number, better reflects effective anti-tumor immunity.

## Simple Summary

Biliary tract cancer (BTC) is a highly aggressive malignancy with a poor prognosis, and effective treatment options remain limited. In this study, we used artificial intelligence-based analysis of pathological images and found that patients with high expression of CD73 in tumor cells showed reduced infiltration of tumor-infiltrating lymphocytes (TILs), including cytotoxic T cells (CTL), in the stroma, which was associated with worse clinical outcomes. These findings suggest that an immunosuppressive tumor microenvironment (TME) mediated by CD73 may contribute to poor prognosis in BTCs. Targeting CD73 may therefore represent a promising therapeutic strategy and provide new treatment opportunities for patients with this disease.

## 1. Introduction

Biliary tract cancer (BTC) is the second leading cause of cancer-related mortality among hepatobiliary and pancreatic malignancies in Japan, following pancreatic cancer, with approximately 20,000 deaths annually [1].

Surgical resection remains the standard first-line treatment for BTC; however, postoperative recurrence is common. For unresectable disease, recent studies have demonstrated the clinical benefit of combining immune checkpoint inhibitors, such as pembrolizumab (anti–PD-1) [2] or durvalumab (anti–PD-L1) [3], with chemotherapy. Nevertheless, objective response rates (ORRs) remain limited to approximately 20–30%.

CD73 is a membrane-bound ectoenzyme that catalyzes the conversion of adenosine monophosphate (AMP) to adenosine and is broadly expressed across various cell types. While extracellular adenosine levels are generally low in normal tissues, they increase under hypoxic or inflammatory conditions and contribute to tissue protection by suppressing inflammation, promoting angiogenesis, and modulating cell–matrix interactions [4,5,6]. In the tumor microenvironment (TME), CD73 has been shown to promote tumor proliferation, angiogenesis, and immune tolerance in several experimental models [7,8]. Overexpression of CD73 has been reported to be associated with aggressive tumor behavior and poor prognosis in several malignancies, including glioma, melanoma, breast, colorectal, pancreatic, and biliary cancers [9,10,11,12,13,14,15,16,17].

Few studies have investigated intratumoral CD73 expression particularly in BTC, and they suggest that high CD73 expression may correlate with lymph node metastasis and poor survival [18]. However, the mechanisms remain unclear.

Meanwhile, immune cells within the TME have emerged as important determinants of prognosis. In many solid cancers, increased infiltration of CD3^+^ or CD8^+^ T cells is associated with favorable outcomes, whereas increased infiltration of regulatory T cells or M2 macrophages is linked to immunosuppression and poor prognosis [19,20,21,22]. Similarly, in BTC, higher CD8^+^ T-cell density has been associated with prolonged overall survival (OS) or disease-free survival (DFS), whereas increased Foxp3^+^ T-cell infiltration correlates with shorter OS [23,24].

Although CD73 expression and immune components of the TME are expected to influence prognosis in BTC, the mechanisms by which CD73 shapes the immune microenvironment including tumor-infiltrating lymphocytes (TILs) and affects clinical outcomes remain unclear. Clarifying the impact of CD73 on immune cell dynamics within the TME and its association with clinical outcomes may provide insights for optimizing therapeutic strategies in BTC.

The present study investigated investigate the relationship between CD73 expression, tumor immune microenvironment features, and the clinical outcomes of BTC.

## 2. Materials and Methods

### 2.1. Patients

This single-center, retrospective, observational cohort study included patients aged ≥18 years with BTC. The inclusion criteria were as follows: (1) Patients who underwent surgery as initial treatment and were pathologically confirmed to have BTC, comprising intrahepatic cholangiocarcinoma, perihilar cholangiocarcinoma, gallbladder cancer, distal cholangiocarcinoma, and ampullary carcinoma, at Hokkaido University Hospital between January 2018 and February 2023, and (2) patients who did not express any withdrawal intention. Exclusion criteria include (1) a history of another malignancy within three years before BTC diagnosis, (2) those deemed unsuitable for inclusion at the discretion of the principal investigator because of insufficient information, and (3) patients who did not provide informed consent to participate in the study by themselves or through a legal representative.

Clinical information was collected from the enrolled patients, including age, sex, physical findings, medical history, diagnosis, prior treatments, treatment details and outcomes, symptoms, performance status, survival data, and family history, as well as pathological findings from the resected specimens.

### 2.2. Specimens

Formalin-fixed, paraffin-embedded blocks prepared from resected primary tumors were sectioned at a thickness of 5 μm. The block containing the largest amount of viable tumor tissue was selected for analysis, whereas specimens with extensive crush artifacts or insufficient tumor volume were excluded. All selected specimens were independently reviewed and verified by two researchers. Hematoxylin and eosin (H&E)-stained slides and unstained sections were prepared, and immunohistochemistry (IHC) was performed on the unstained sections.

### 2.3. Immunohistochemistry

Eligible specimens were subjected to IHC staining using a rabbit monoclonal anti-CD73 antibody (#13160, Cell Signaling Technology, Danvers, MA, USA) diluted 1:100, a rabbit polyclonal anti-CD3 antibody (Dako #IR503, Agilent, Santa Clara, CA, USA), a mouse monoclonal anti-CD8 antibody (Dako #IR623, Agilent, Santa Clara, CA, USA), a mouse monoclonal anti-CD163 antibody (#CD163-L-CE, Leica Biosystems, Nussloch, Germany) diluted 1:500, and a mouse monoclonal anti-Foxp3 antibody (#ab20034, abcam, Cambridge, UK) diluted 1:100. CD3^+^, CD8^+^, and Foxp3^+^ lymphocytes and CD163^+^ immune cells were considered to represent mature T lymphocytes, cytotoxic T cells (CTLs), regulatory T cells (Tregs), and M2 macrophages, respectively.

Visualization was performed using the horseradish peroxidase (HRP)-labeled polymer method with a secondary antibody (Dako #SM802, EnVision FLEX/HRP goat secondary antibody molecules, Agilent, Santa Clara, CA, USA).

### 2.4. AI-Based Image Analysis and Quantification

All H&E and IHC slides were digitized into whole slide image (WSI) files at 20× magnification using a NanoZoomer 2.0HT Digital slide scanner (Hamamatsu Photonics K.K., Shizuoka, Japan), 0 and the tumor bed was defined within each H&E-WSI.

To quantify the density of lymphocytes within both the tumor epithelium and the stroma, we utilized an AI-powered pathological image analysis system, DeepPathFinder™ (biomy Inc., Tokyo, Japan). The system incorporates deep learning-based segmentation models specifically optimized for the detection of epithelial regions and lymphocytes in histopathological images.

The AI models were trained and validated using the SegPath dataset [25], a large-scale, standardized dataset for cancer histology segmentation. To ensure robustness, the dataset was split into training, validation, and test sets following the protocols described by Komura et al. [25]. Specifically, the epithelium segmentation model was developed using 21,912 training images, 2259 validation images, and 2338 test images. The lymphocyte detection model was trained on 10,453 images, with 1082 and 738 images used for validation and testing, respectively.

In this study, the “tumor bed” on the WSIs was first defined by a gastroenterologist (S.S.) and a pathologist (K.C.H.), both blinded to clinical data and patient outcomes.

The tumor bed was defined using the following procedure: First, H&E-stained slides of surgical specimens were examined under a light microscope to manually outline the approximate tumor region, which was then digitized into the WSIs. Within each WSI, the area of the deepest invasion of tumor cells was thoroughly identified, and the tumor boundary was re-demarcated to define the tumor bed, which served as the representative region of the TME.

**Figure 1:**
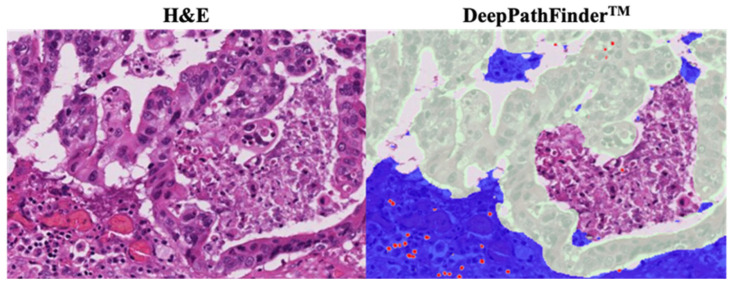
Representative hematoxylin and eosin (H&E) stained image and corresponding DeepPathFinder™-based image analysis (original magnification approximately ×200). The original H&E image (**left**) and (the DeepPathFinder™-generated annotation map (**right**) shows automated segmentation of tumor (green) and stromal (blue) regions and detection of lymphocytes (red) within the tumor bed.

Within this defined tumor bed, regions predicted as epithelium by the AI were classified as “tumor epithelium,” while the remaining tissue areas were defined as “stroma.” The trained models were then applied to the WSIs to quantify the density of lymphocytes within these defined tumor epithelial and stromal compartments (Figure 1) [26,27]. Lymphocytes within the tumor bed were defined as tumor-infiltrating lymphocytes (TILs).

Furthermore, DeepPathFinder™ was employed to quantify positive staining areas in IHC images. To ensure spatial correspondence between morphological features and protein expression, non-rigid image registration was performed to align the H&E-stained WSIs with their corresponding IHC-stained WSIs to a common coordinate system. Positive staining areas on the IHC images were identified via digital image analysis using intensity thresholds established by a pathologist (K.C.H.). Finally, the density of these positive areas was calculated within the tumor epithelium and stromal compartments, as defined on the co-registered H&E images [28].

In this study, DeepPathFinder™ was used to evaluate CD73 expression and to detect CD3^+^ lymphocytes, CD8^+^ lymphocytes, Foxp3^+^ lymphocytes, and CD163^+^ immune cells in both tumor and stromal areas. CD73 immunostaining was predominantly localized to the cell membrane of tumor cells, with occasional cytoplasmic staining. Stromal staining was generally weaker and more heterogeneous.

**Figure 2:**
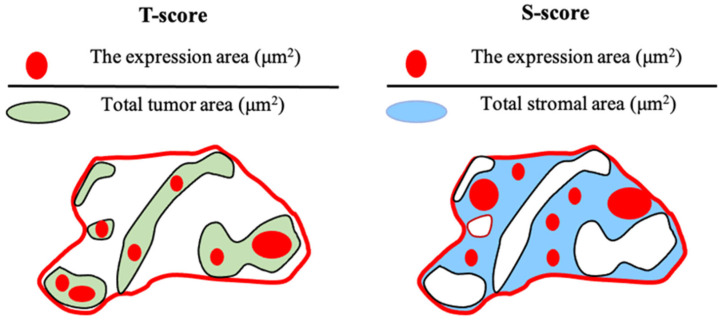
Schematic illustration of the T-score and S-score. The tumor bed was divided into tumor and stromal compartments, and the T-score and S-score represent the quantitative measurements of CD73 (CD3, CD8, Foxp3, and CD163) expression or lymphocyte infiltration within each compartment, respectively.

To evaluate TILs, infiltrating lymphocytes within the tumor and stromal areas identified on H&E-WSI were quantified as the area of lymphocytes per unit area, and defined as the T-TIL and S-TIL scores, respectively (Figure 2). These were dichotomized into high and low groups based on the median cutoff value.

**Figure 3:**
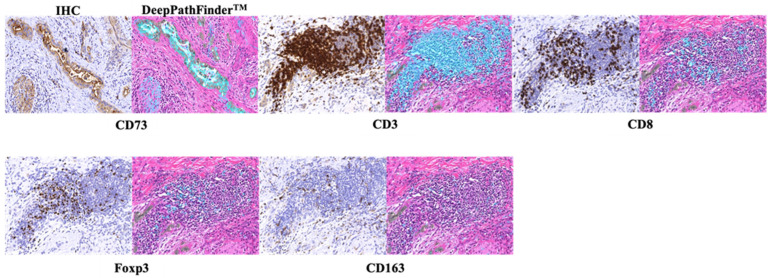
Representative IHC staining images (**left**) and corresponding AI-based analysis images generated by DeepPathFinder™ (**right**) for CD73, CD3, CD8, Foxp3, and CD163 (original magnification approximately ×200). IHC-positive areas (cyan) were automatically identified and overlaid onto tumor regions (green) delineated on the corresponding H&E–stained images, enabling quantitative assessment of IHC-positive area within the tumor compartment.

Similarly, for the evaluation of CD73 expression and each immune cell subset (CD3^+^, CD8^+^, Foxp3^+^, and CD163^+^ cells), the area of positive immunostaining within the tumor and stromal regions was quantified per unit area and defined as T-CD73/CD3/CD8/Foxp3/CD163 and S-CD73/CD3/CD8/Foxp3/CD163 scores, respectively (Figure 3).

The area of the tumor bed, the validity of immunostaining, and the accuracy of CD73+ and lymphocyte detection by DeepPathFinder™ were independently reviewed and validated by a gastroenterologist (S.S.) and a pathologist (K.C.H.), both blinded to clinicopathological data and patient outcomes.

### 2.5. Outcome Measurements

The primary outcome was the association between CD73 expression and OS, defined as the time from surgery to death due to any cause. Secondary outcomes included the association between TILs and OS, the association between CD73 expression and TILs, and the association between CD73 expression and immune cell infiltration. For OS analyses, patients were classified into high and low groups according to the median T-CD73, S-CD73, T-TIL, and S-TIL scores.

### 2.6. Statistical Analysis

All statistical analyses were performed using EZR version 1.68 (Saitama Medical Center, Jichi Medical University, Saitama, Japan) [29], which is a graphical user interface for R (version 4.3.2; R Foundation for Statistical Computing, Vienna, Austria).

OS was estimated using the Kaplan–Meier method with 95% confidence intervals (CIs), and the differences between groups were assessed using the log-rank test. Hazard ratios (HRs) were calculated using the Cox proportional hazards regression analysis.

Univariable Cox proportional hazards analyses were performed to identify potential prognostic factors associated with OS. Variables with a *p*-value < 0.10 in the univariable analyses were subsequently included in the multivariable Cox proportional hazards model to adjust for potential confounding factors in patients with BTC.

Correlations between CD73 expression and TILs, CD3^+^ lymphocytes, CD8^+^ lymphocytes, Foxp3^+^ lymphocytes, and CD163^+^ immune cells were evaluated using Spearman’s rank correlation coefficient. Statistical significance was set at *p* < 0.05.

## 3. Results

### 3.1. Clinicopathological Characteristics

**Figure 4:**
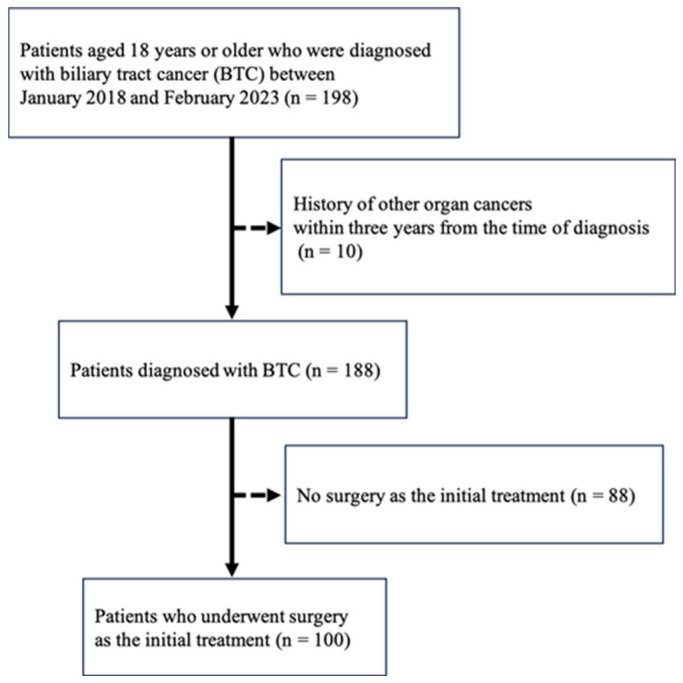
Flowchart of this study.

One hundred patients with biliary tract cancer who underwent curative surgery as the initial treatment were enrolled in this study (Figure 4).

The primary tumor sites comprised intrahepatic cholangiocarcinoma (IHCC) in 3 patients (3%), perihilar cholangiocarcinoma (PHCC) in 43 (43%), gallbladder cancer (GBC) in 12 (12%), distal cholangiocarcinoma (DBC) in 26 (26%), and ampullary carcinoma (AC) in 16 (16%). According to the Union for International Cancer Control (UICC) 8th edition TNM classification, 1 patient (1%) was stage 0, 20 (20%) were stage I, 44 (44%) were stage II, 21 (21%) were stage III, and 14 (14%) were stage IV. During the observation period, 38 deaths were observed. Lymph node and/or distant metastasis was present in 41 patients. R0 resection was achieved in 78 patients, whereas 22 patients underwent R1 resection. Adjuvant chemotherapy was administered to 24 patients (Table 1).

### 3.2. Analysis of Immunohistochemistry with DeepPathFinder™

The median values and ranges of the CD73, TIL, CD3, CD8, Foxp3, and CD163 scores are shown in Table 2. The CD73 score was higher in the tumor compartment than in the stroma, whereas the TIL score was higher in the stromal compartment than in the tumor compartment (Supplementary Table S1).

### 3.3. Impact of CD73 and TILs on Overall Survival

Patients were divided into high and low groups according to the median T-CD73, S-CD73, T-TIL, and S-TIL scores.

First, clinicopathological characteristics were compared between the low- and high-CD73-score groups. Patients with a high T-CD73 score had a significantly higher frequency of lymph node metastasis compared with those with a low T-CD73 score. In contrast, no significant differences in clinicopathological characteristics were observed between the low and high S-CD73 score groups (Table 2).

Regarding TIL scores, comparison of clinicopathological characteristics between the low and high groups showed that patients with a low T-TIL score had a significantly higher frequency of lymph node metastasis. In contrast, patients with a low S-TIL score more frequently had advanced T stage and lymph node metastasis (Table 3).

**Figure 5:**
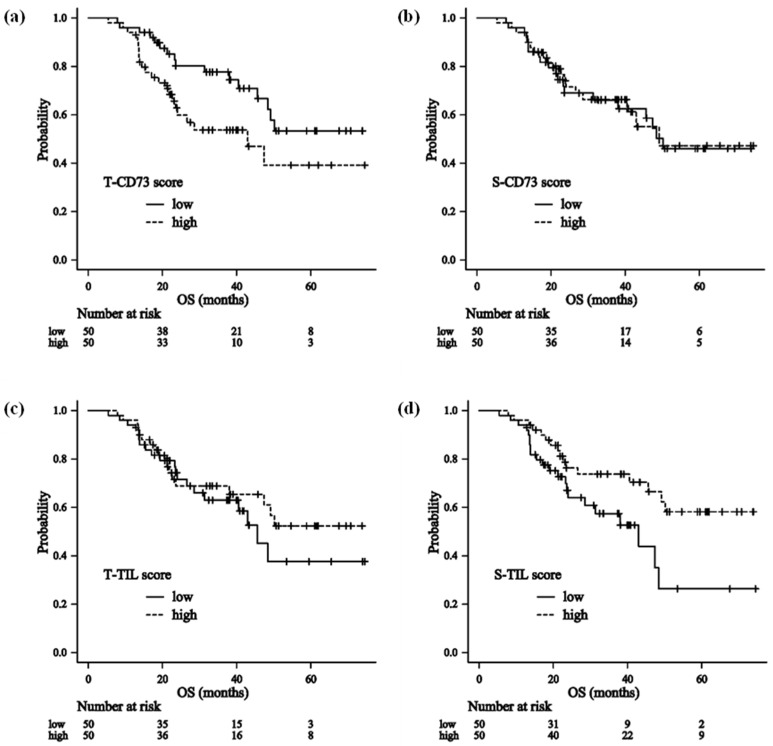
Effect of the CD73 and TIL scores. Kaplan–Meier curves of the overall survival in 100 patients (**a**) 50 in the T-CD73-high group and 50 in the T-CD73-low group (**b**) 50 in the S-CD73-high group and 50 in the S-CD73-low group (**c**) 50 in the T-TIL-high group and 50 in the T-TIL-low group (**d**) 50 in the S-TIL-high group and 50 in the S-TIL-low group.

OS was compared between the two groups for each item score as follows: T-CD73 score: median OS 43.0 months (95% CI, 22.7–NR) in the high group vs. NR (95% CI, 45.6–NR) in the low group (HR 1.97; 95% CI, 1.03–3.79; *p* = 0.041) (Figure 5a). S-CD73 score: 49.1 months (95% CI, 40.5–NR) vs. 50.2 months (95% CI, 31.3–NR) (HR 0.96; 95% CI, 0.51–1.82; *p* = 0.898) (Figure 5b). T-TIL score: NR (95% CI, 38.1–NR) vs. 45.6 months (95% CI, 28.6–NR) (HR 0.78; 95% CI, 0.41–1.49; *p* = 0.456) (Figure 5c). S-TIL score: NR (95% CI, 45.6–NR) vs. 43.0 months (95% CI, 23.0–48.4) (HR 0.49; 95% CI, 0.25–0.94; *p* = 0.032) (Figure 5d).

In the univariable analysis, lymph node status, distant metastasis status, resection margin status (R0/R1), T-CD73 score (high/low), and S-TIL score (high/low) were significantly associated with OS. Furthermore, to evaluate the association between T-CD73 score and clinicopathological factors as well as prognosis, a multivariate analysis was performed including variables with *p* < 0.1 in the univariate analysis as covariates, which identified sex, distant metastasis status, resection margin status (R0/R1), and T-CD73 score as independent prognostic factors (Table 4).

### 3.4. Correlation Between CD73 and TILs

**Figure 6:**
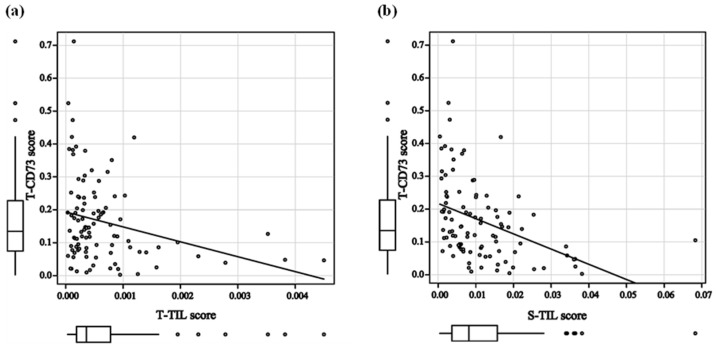
Correlations between T-CD73 and TIL scores/immune cell subsets. Spearman’s rank correlation coefficient between (**a**) T-CD73 and T-TIL scores and (**b**) T-CD73 and S-TIL scores.

Spearman’s rank correlation coefficient showed that T-CD73 scores were negatively correlated with both T-TIL (r = −0.264; *p* = 0.008) and S-TIL scores (r = −0.495; *p* < 0.001) (Figure 6). Meanwhile, the S-CD73 score was weakly correlated with the S-TIL score (r = −0.208; *p* = 0.038) but not with the T-TIL score (r = −0.191; *p* = 0.057) (Supplementary Figure S1).

### 3.5. Correlation Between CD73 and Immune Cell Subsets

**Figure 7:**
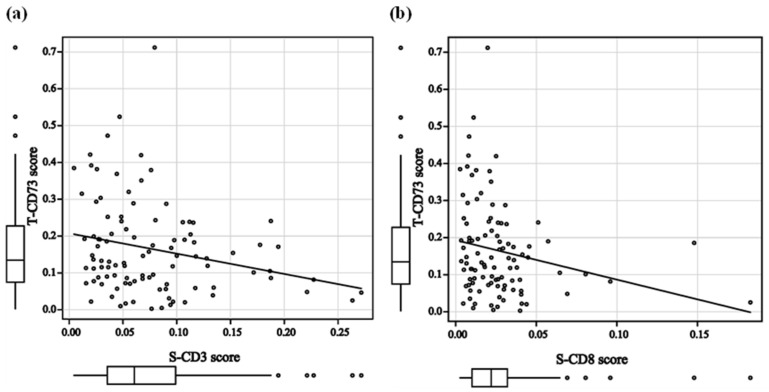
Correlations between T-CD73 and immune cell subsets. Spearman’s rank correlation coefficient between (**a**) T-CD73 and S-CD3 scores and (**b**) T-CD73 and S-CD8 scores.

The T-CD73 score was not significantly correlated with the T-CD3 (r = −0.136; *p* = 0.180), T-CD8 (r = −0.174; *p* = 0.084), T-Foxp3 (r = −0.081; *p* = 0.423), or T-CD163 scores (r = −0.012; *p* = 0.909) (Supplementary Figure S2). In contrast, the T-CD73 scores was negatively correlated with S-CD3 (r = −0.246; *p* = 0.014) and S-CD8 scores (r = −0.266; *p* = 0.008) (Figure 7), while no significant correlation was observed with S-Foxp3 (r = −0.081; *p* = 0.421) and S-CD163 scores (r = −0.034; *p* = 0.734) (Supplementary Figure S3), indicating an inverse relationship between intratumoral CD73 expression and stromal mature T lymphocyte/CTL density.

The S-CD73 score was not significantly correlated with T-CD3 (r = −0.051; *p* = 0.620), T-CD8 (r = 0.012; *p* = 0.909), T-Foxp3 (r = 0.138; *p* = 0.171), or T-CD163 scores (r = 0.085; *p* = 0.399). The correlations between the S-CD73 and S-CD3 (r = 0.067; *p* = 0.508), S-CD8 (r = 0.016; *p* = 0.873), and S-Foxp3 scores (r = 0.141; *p* = 0.162) were also not significant, whereas a positive correlation was observed between the S-CD73 and S-CD163 scores (r = 0.317; *p* = 0.001) (Supplementary Figure S4).

## 4. Discussion

Using AI-based tumor microenvironment analysis, this study revealed that high tumoral CD73 expression causes poor prognosis due to immunosuppression mediated by stromal lymphocytes, rather than by those in the tumor.

No studies have investigated the correlation between CD73 expression and stromal TILs or their association with BTC prognosis. Using DeepPathFinder™, this study has clearly delineated tumor and stromal regions within the TME, allowing for a more precise quantification of CD73 expression and TIL density in each compartment. Importantly, this study demonstrated for the first time that increased CD73 expression is inversely correlated with stromal TIL density, thereby providing mechanistic insights into how CD73 upregulation may contribute to poor prognosis through the suppression of stromal immune infiltration.

TILs are considered important prognostic indicators whose location, density, and functional orientation within the TME define their prognostic and predictive values [30]. To date, evaluation of TILs on H&E-WSIs has focused on stromal TILs, as intratumoral TILs exhibit marked spatial heterogeneity and are often difficult to identify, and therefore stromal TIL assessment is recommended [31]. In contrast, the prognostic value of TILs in BTC has not been consistently reported. In BTC, a higher stromal TIL density was associated with prolonged recurrence-free and OS after resection, whereas intratumoral TILs had lower prognostic value [32,33,34].

In this study, the use of DeepPathFinder™, an AI-based image analysis software, enabled not only accurate quantification of stromal TILs but also reliable measurement of intratumoral TILs. Our results showed that intratumoral TIL density within the tumor microenvironment was not associated with prognosis, whereas higher stromal TIL density was associated with better survival. These findings are consistent with previous reports and suggest that lymphocyte density within the stroma contributes more substantially to antitumor immunity and may serve as a useful prognostic predictor in BTC.

Several BTC studies have focused on specific immune cell subsets within the stromal TIL populations. Several studies have indicated that a higher number or density of CD8^+^ T cells around cancer cells was associated with longer OS or DFS, whereas increased Foxp3^+^ T-cell infiltration was linked to shorter OS [23,34].

Consistent with these observations, our correlation analysis showed that tumoral CD73 expression was inversely associated with CD3^+^ and CD8^+^ T-cell densities, implying the selective suppression of stromal CTL infiltration, although no significant association was observed with Foxp3^+^ T cells or CD163^+^ macrophages.

These results suggest that increased CD73 activity in tumor cells within the TME reduces the density of stromal CTLs and other TILs, thereby promoting immune escape of tumor cells and contributing to poor prognosis.

Although H-score is commonly used in clinical practice to evaluate IHC expression in tumor cells [35], its reproducibility and objectivity remain limited. DeepPathFinder™ overcomes these issues by recognizing the entire tumor region and calculating IHC-positive areas using a uniform, non-variable analytical standard, thereby ensuring consistent, reproducible, and objective measurements. Furthermore, this approach enables simultaneous evaluation of immune-related markers such as CD3, CD8, Foxp3, and CD163, providing a comprehensive assessment of immune cell populations within the tumor microenvironment and enhancing our understanding of tumor immunity.

The present study has some limitations. First, this was a retrospective, single-center study; therefore, a large cohort prospective study is required to validate these findings. Second, the site and stage of BTC could not be investigated because of the small number of patients in each cohort. Third, the TIL score is evaluated on H&E-stained slides by identifying individual lymphocytes and calculating their total area, whereas the scores for each immune cell type are based on the total immunostained area within the tumor or stroma. Because immunostained regions are identified more broadly rather than at the single-cell level, these areas tend to be larger, which may account for the discrepancy between the TIL score and the immune cell scores. Forth, the T-CD73 score is calculated as the density of CD73-positive area in tumor regions and therefore does not capture intratumoral heterogeneity.

Future studies should include large-scale, multicenter prospective trials to validate the findings of the present study. In addition, detailed analysis of the TME using spatial transcriptomics is warranted to clarify the immunomodulatory effects of CD73 and to contribute to the expansion of therapeutic options and improved outcomes for patients with BTC.

## 5. Conclusions

High tumoral CD73 levels promote immune escape by reducing stromal CTL infiltration, leading to poorer survival outcomes in BTC. These findings suggest that CD73 is a key factor driving immunosuppression and support the therapeutic rationale for targeting the CD73–adenosine pathway.

## Figures and Tables

**Table 1:** Patient characteristics.

	Total n = 100
Age, year—median (range)	72 (27–87)
Sex, n	
Male/Female	72/28
Primary tumor location, n	
IHCC/PHCC/GBC/DCC/AC	3/43/12/26/16
Clinical stage *, n	
0/I/II/III/IV	1/20/44/21/12
T stage, n	
Tis/T1/T2/T3/T4	1/20/56/18/5
N stage, n	
N0/N1, 2	61/39
M stage, n	
M0/M1	89/11
Residual tumor, n	
R0/R1	78/22
Adjuvant therapy, n	
Yes/No	24/76
Number of deaths, n	38

* UICC, Union for International Cancer Control classification of malignant tumors, 8th edition; IHCC, intrahepatic cholangiocarcinoma; PHCC, perihilar cholangiocarcinoma; GBC, gallbladder carcinoma; DCC, distal cholangiocarcinoma; AC, ampullary carcinoma.

**Table 2:** Patient characteristics according to T- and S-CD73 score (low vs. high).

	T-CD73 Score			S-CD73 Score		
Characteristics	Low (n = 50)	High (n = 50)	*p*-Value	Low (n = 50)	High (n = 50)	*p*-Value
Age, year—median (range)	72 (27–87)	72 (27–83)	0.852	71 (54–83)	72 (27–87)	0.712
Sex, n						
Male/Female	35/15	37/13	0.824	38/12	34/16	0.504
Primary tumor location, n						
IHCC/PHCC/GBC/DCC/AC	2/16/9/13/10	1/27/3/13/6	0.128	2/23/7/13/5	1/20/5/13/11	0.537
Clinical stage *, n						
0/I/II/III/IV	1/15/22/7/5	0/5/22/14/9	0.050	0/13/20/11/6	1/7/24/10/8	0.478
T stage, n						
Tis/T1/T2/T3/T4	1/15/25/7/2	0/5/31/11/3	0.102	0/14/25/9/2	1/6/31/9/3	0.283
N stage, n						
N0/N1, 2	37/13	24/26	0.013	35/15	26/24	0.100
M stage, n						
M0/M1	45/5	44/6	1.000	44/6	45/5	1.000
Residual tumor, n						
R0/R1	40/10	38/12	0.810	38/12	40/10	0.810
Adjuvant therapy, n						
Yes/No	10/40	14/36	0.483	13/37	11/39	0.815
Number of deaths, n	16	22	0.303	20	18	0.837

* UICC, Union for International Cancer Control classification of malignant tumors, 8th edition; IHCC, intrahepatic cholangiocarcinoma; PHCC, perihilar cholangiocarcinoma; GBC, gallbladder carcinoma; DCC, distal cholangiocarcinoma; AC, ampullary carcinoma.

**Table 3:** Patient characteristics according to T- and S-TIL score (low vs. high).

	T-TIL Score			S-TIL Score		
Characteristics	Low (n = 50)	High (n = 50)	*p*-Value	Low (n = 50)	High (n = 50)	*p*-Value
Age, year—median (range)	72 (27–84)	71.5 (50–87)	0.948	71 (27–82)	72 (50–87)	0.188
Sex, n						
Male/Female	33/17	39/11	0.265	39/11	33/17	0.265
Primary tumor location, n						
IHCC/PHCC/GBC/DCC/AC	1/20/5/15/9	2/23/7/11/7	0.783	2/23/3/14/8	1/20/9/12/8	0.449
Clinical stage *, n						
0/I/II/III/IV	0/7/22/13/8	1/13/22/8/6	0.370	0/5/23/13/9	1/15/21/8/5	0.077
T stage, n						
Tis/T1/T2/T3/T4	0/7/29/11/3	1/13/27/7/2	0.411	0/7/24/15/4	1/13/32/3/1	0.004
N stage, n						
N0/N1, 2	24/26	37/13	0.013	25/25	36/14	0.040
M stage, n						
M0/M1	43/7	46/4	0.525	44/6	45/5	1.000
Residual tumor, n						
R0/R1	38/12	40/10	0.810	35/15	43/7	0.090
Adjuvant therapy, n						
Yes/No	16/34	8/42	0.100	16/34	8/42	0.815
Number of deaths, n	20	18	0.837	22	16	0.303

* UICC, Union for International Cancer Control classification of malignant tumors, 8th edition; IHCC, intrahepatic cholangiocarcinoma; PHCC, perihilar cholangiocarcinoma; GBC, gallbladder carcinoma; DCC, distal cholangiocarcinoma; AC, ampullary carcinoma.

**Table 4:** Univariable and multivariable Cox proportional hazards analyses for prognostic factors in patients with biliary tract cancer.

		Univariate Analysis		Multivariate Analysis	
Characteristics		HR (95%CI)	*p*-Value	HR (95%CI)	*p*-Value
Age, year	70<	1.023 (0.523–2.001)	0.947		
Sex	Male	Reference		Reference	
	Female	0.397 (0.155–1.02)	0.055	0.272 (0.103–0.719)	0.008
T stage	Tis, T1, T2	Reference			
	T3, T4	1.707 (0.817–3.568)	0.155		
N stage	N0	Reference		Reference	
	N1, N2	2.110 (1.114–3.997)	0.022	1.553 (0.783–3.082)	0.208
M stage	M0	Reference		Reference	
	M1	4.106 (1.806–9.334)	<0.001	3.350 (1.370–8.192)	0.008
Resection margin	R0	Reference		Reference	
	R1	2.777 (1.426–5.406)	0.003	2.417 (1.223–4.777)	0.011
Adjuvant therapy	No	Reference			
	Yes	0.833 (0.345–2.012)	0.685		
T-CD73score	Low	Reference		Reference	
	High	1.973 (1.027–3.790)	0.041	2.227 (1.140–4.349)	0.019
S-TILscore	Low	Reference			
	High	0.485 (0.25–0.94)	0.032		

T-, tumor-; S-, stroma-; TIL, tumor-infiltrating lymphocyte; HR, hazard ratio; CI, confidence interval.

## Data Availability

The data presented in this study are available on request from the corresponding author. The data are not publicly available due to institutional data policy.

## Supplementary Materials

The following supporting information can be downloaded at https://www.mdpi.com/article/10.3390/cancers18060975/s1. Table S1: T- and S-score of parameters; Figure S1: Spearman’s rank correlation coefficient between S-CD73 and TIL scores; Figure S2: Spearman’s rank correlation coefficient between T-CD73 and intratumoral immune cell subsets; Figure S3: Spearman’s rank correlation coefficient between T-CD73 and stromal immune cell subsets; Figure S4: Spearman’s rank correlation coefficient between S-CD73 and intratumoral/stromal immune cell subsets.

## Abbreviations

The following abbreviations are used in this manuscript:

AI	Artificial intelligence
AMP	Adenosine monophosphate
BTC	Biliary tract cancer
CD	Cluster of differentiation
CTL	Cytotoxic T lymphocyte
DFS	Disease-free survival
FFPE	Formalin-fixed, paraffin-embedded
Foxp3	Forkhead box P3
H&E	Hematoxylin and eosin
HR	Hazard ratio
HRP	Horseradish peroxidase
IHC	Immunohistochemistry
OS	Overall survival
PD-1	Programmed cell death protein 1
PD-L1	Programmed death ligand 1
S-1	Tegafur-gimeracil-oteracil potassium
TIL	Tumor-infiltrating lymphocyte
TME	Tumor microenvironment
TNM	Tumor, node, metastasis
Treg	Regulatory T cell
UICC	Union for International Cancer Control
