# Supplementary material for: High CD73 Expression Is Associated with Poor Prognosis in Biliary Tract Cancer Through Reduced Stromal Tumor-Infiltrating Lymphocytes
Source: Cancers (Basel). 2026 Mar 18;18(6):975. doi: 10.3390/cancers18060975 (PMC13025982; doi:10.3390/cancers18060975)
Supplement: Supplementary file 1 [file cancers-18-00975-s001.zip › cancers-4195184-supplementary.pdf]

Table S1. T- and S-score of parameters

	Median (×10^{-4}, range)	
	**T-score**	**S-score**
CD73	1346 (28.1–7120)	716.7 (65.5–3812)
CD3	637.7 (7.5–3864)	639.2 (44.4–2716)
CD8	146.2 (0.82–1637)	222.0 (9.6–1828)
Foxp3	19.2 (0.47–626.7)	27.5 (1.0–415.9)
CD163	237.9 (10.8–4809)	380.9 (37.2–2119)
TIL	3.6 (0.33–45.0)	81.7 (4.4–682.6)

T-, tumor-; S-, stroma-; TIL, tumor-infiltrating lymphocyte

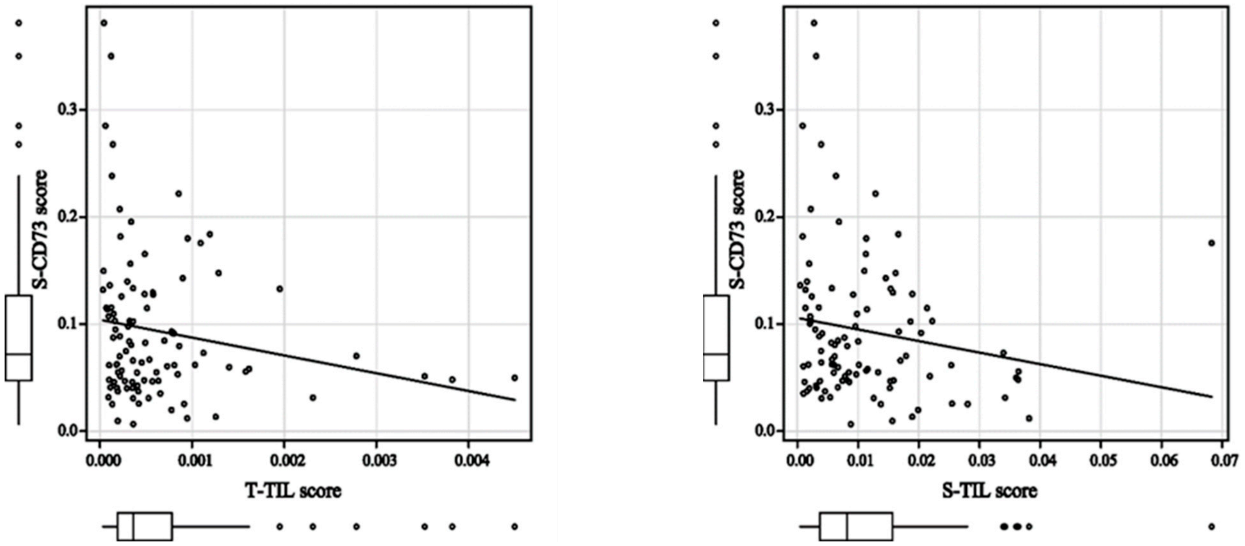

(a) **(b)**

Figure S1. Spearman's rank correlation coefficient between S-CD73 and TIL scores. These figures present the correlations between (a) S-CD73 and T-TIL scores and (b) S-CD73 and S-TIL scores. The S-CD73 score was weakly correlated with the S-TIL score (r = –0.208; p = 0.038) but not with the T-TIL score (r = –0.191; p = 0.057).

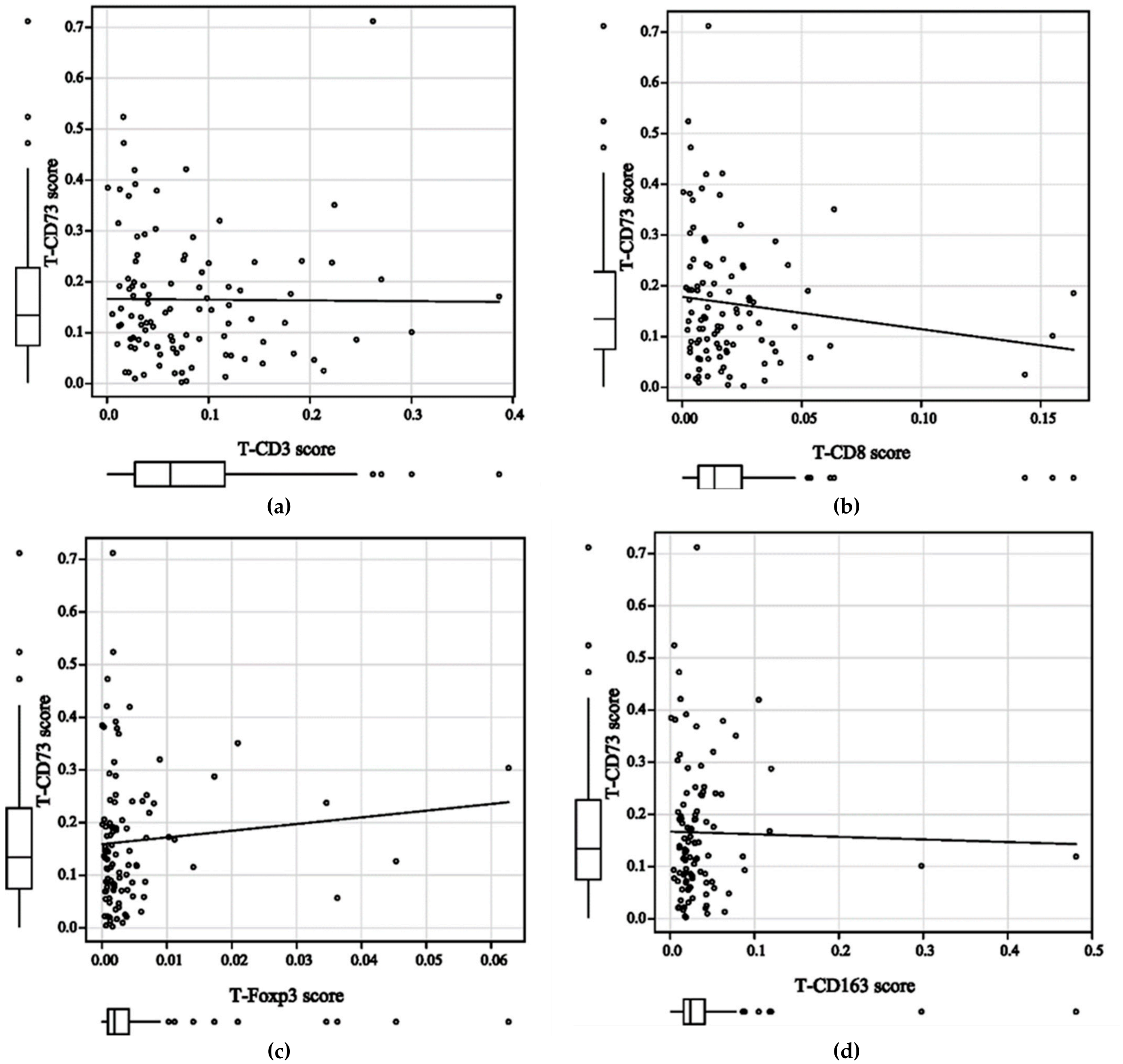

Figure S2. Spearman's rank correlation coefficient between T-CD73 and intratumoral immune cell subsets. These figures present the correlations between (a) T-CD73 and T-CD3 scores, (b) T-CD73 and T-CD8 scores, (c) T-CD73 and T-Foxp3 scores, and (d) T-CD73 and T-CD163 scores. No significant correlations were observed between the T-CD73 score and any intratumoral immune cell subsets.

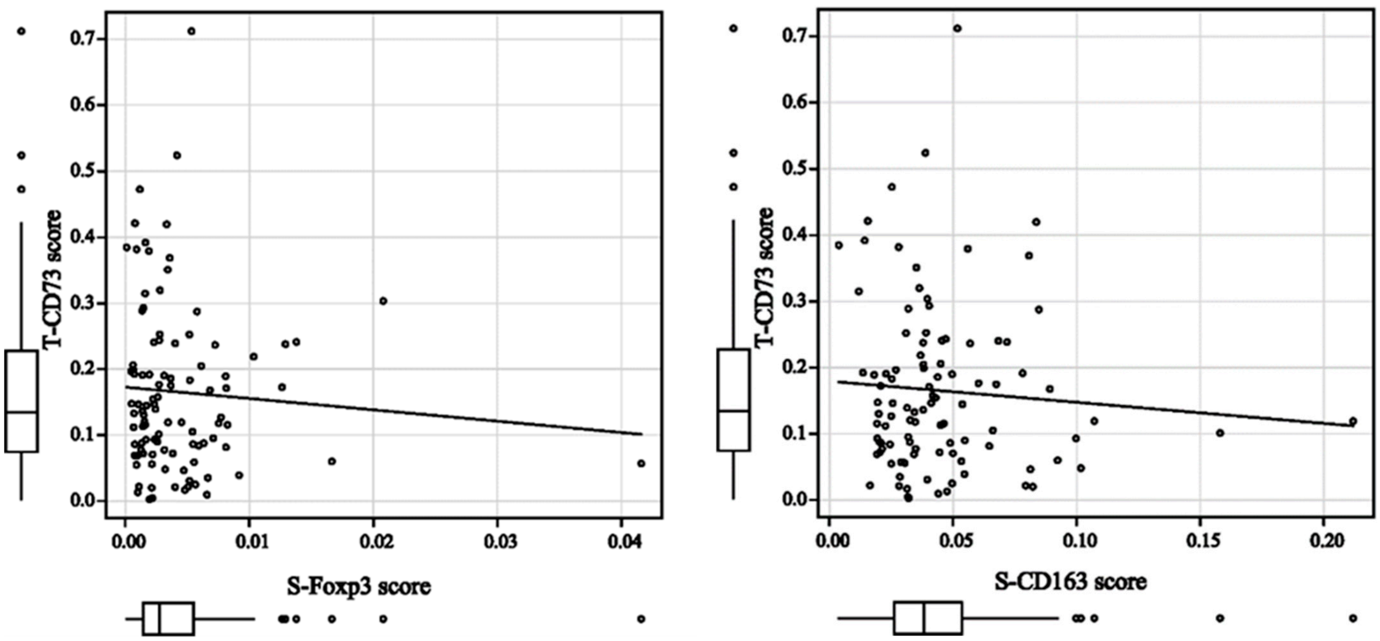

(a) **(b)**

Figure S3. Spearman's rank correlation coefficient between T-CD73 and stromal immune cell subsets. These figures present the correlations between (a) T-CD73 and S-Foxp3 scores, (b) T-CD73 and S-CD163 scores. No significant correlation was observed with S-Foxp3 (r = –0.081; p = 0.421) and S-CD163 scores (r = –0.034; p = 0.734).

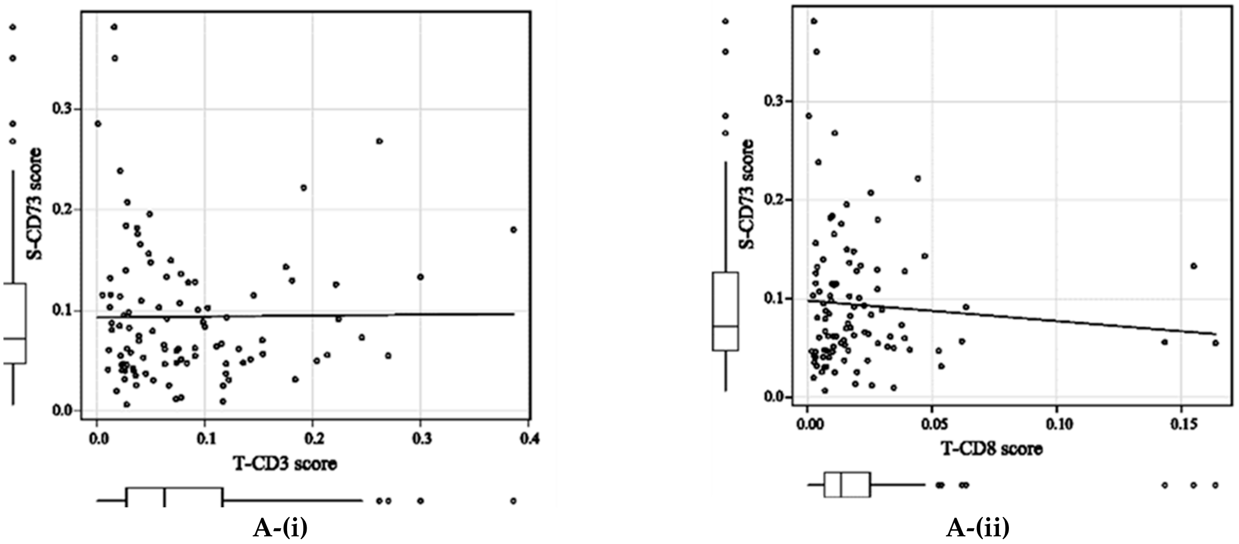

A-(i) **A-(ii)**

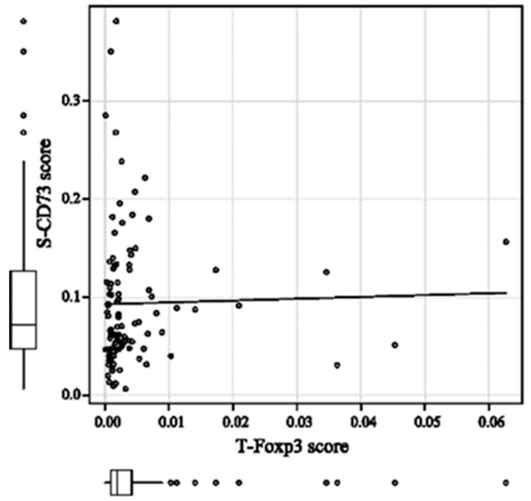

A-(iii)

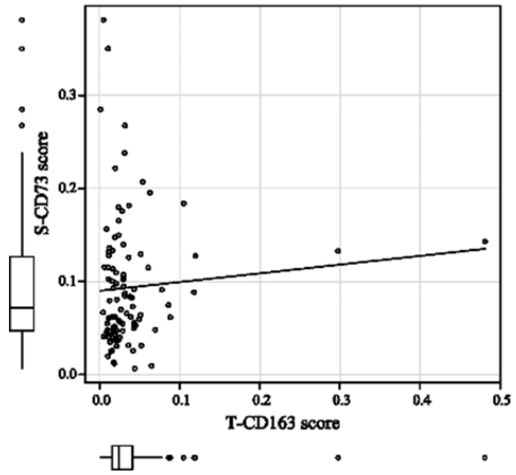

A-(iv)

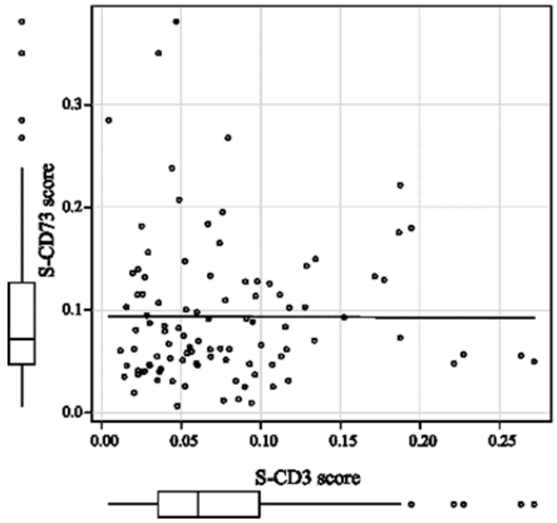

B-(i)

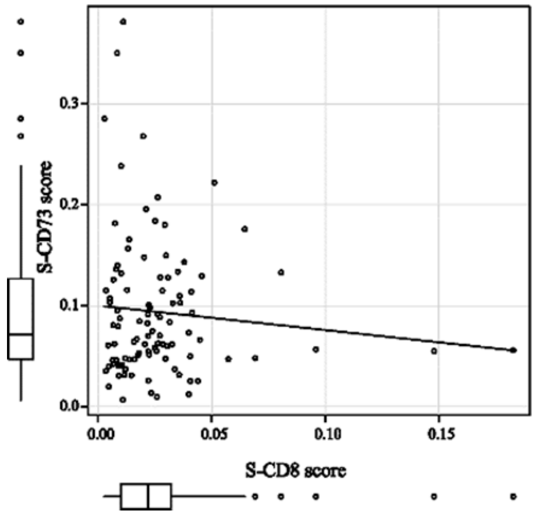

B-(ii)

Figure S4. Spearman's rank correlation coefficient between S-CD73 and intratumoral/stromal immune cell subsets. These figures present the correlations between (A) intratumoral and (B) stromal immune cell subsets. The upper panel (A) shows correlations between the S-CD73 score and intratumoral immune cell subsets: (i) S-CD73 score and T-CD3 score, (ii) S-CD73 score and T-CD8 score, (iii) S-CD73 score and T-Foxp3 score, and (iv) S-CD73 score and T-CD163 score. The lower panel (B) shows correlations between the S-CD73 score and stromal immune cell subsets: (i) S-CD73 score and S-CD3 score, (ii) S-CD73 score and S-CD8 score, (iii) S-CD73 score and S-Foxp3 score, and (iv) S-CD73 score and S-CD163 score.
